# The Impact of Climate Change on the Spatial Distribution of Seven *Meconopsis* Species in China: A MaxEnt Model‐Based Predictive Analysis

**DOI:** 10.1002/ece3.73824

**Published:** 2026-06-17

**Authors:** Yuanzhao Yang, Jiao Ma, Xinyu Chen, Lu Zhang, Pei Tu, Yuanzhi Pan

**Affiliations:** ^1^ Chengdu Botanical Garden (Chengdu Park City Botanical Science Research Institute) Chengdu P. R. China; ^2^ Horticulture Research Institute Sichuan Academy of Agricultural Sciences Chengdu P. R. China; ^3^ College of Forestry Sichuan Agricultural University Chengdu P. R. China

**Keywords:** environmental variables, MaxEnt model, *Meconopsis* spp., potential suitable distribution

## Abstract

Plant distribution correlates with climate change, especially in mountain flora. Using an optimized MaxEnt model and the latest WorldClim 2.1 bioclimatic data, this study identified the key environmental determinants of habitat suitability and projected the current and future distribution patterns of seven *Meconopsis* spp. in China under various climate change scenarios. Results reveal that the primary environmental variables shaping the distribution are isothermal (bio3), seasonal variation coefficient of temperature (bio4), and elevation. Seven taxa were concentrated in southwestern China, with suitable habitats encompassing 1.3% to 10.1% of the land area in China. Under future climate scenarios, *M. lyrata* is expected to experience a decline in habitat suitability, and other species showed mixed responses. Given the limited extent of suitable habitats for most *Meconopsis* species in China, ex‐situ conservation—particularly through initiatives spearheaded by national botanical research institutions—should prioritize domestication and conservation efforts to safeguard genetic diversity.

## Graphical abstract

We addressed this gap by evaluating the current and future distribution of eight *Meconopsis* species using optimized MaxEnt modeling and the WorldClim 2.1 bioclimatic datasets. Our research provides critical insights into the divergent fates of these species under future climate scenarios. Specifically, we found that Bio3, Bio4, and elevation are the key determinants of *Meconopsis* distribution. Climate change is projected to cause disproportionate effects on *Meconopsis* distribution.

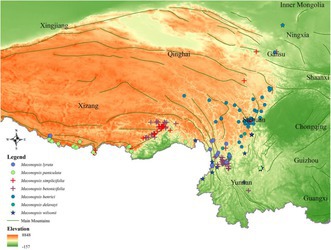

## 1 Introduction

Climate strongly influences plant distribution, community composition, and biodiversity (Walther et al. 2002). Mountain flora (alpine plants) are particularly sensitive to climatic shifts, with research indicating that changes in temperature and precipitation can drive both range contractions and expansions (Vanneste et al. 2017; Cai et al. 2021). In China, future climate projections indicate overall warming trends and increased humidity (Chao et al. 2023), along with increasing frequency and severity of extreme weather events (Wang et al. 2022), according to reports of WMO (World Meteorological Organization) 2024 was the hottest year on record. These trends pose significant threats to the survival of mountain flora species. Furthermore, alpine plants have adapted to the large temperature difference over their long‐term evolution. Whereas global warming would induce that nighttime temperatures are rising more rapidly and reducing the diurnal temperature range. This constitutes a potential risk for alpine flora. Therefore, understanding how climate variability affects species distribution and migration patterns is essential for advancing risk assessments and developing effective conservation strategies for vulnerable taxa.

Species distribution modeling techniques such as MaxEnt (maximum entropy), GLM (generalized linear model), GARP (genetic algorithm for rule‐set prediction), BIOCLIM (bioclimatic envelope model), and ENFA (ecological niche factor analysis) have been widely used to predict the potential effects of climate change on plant species distribution (Zhang et al. 2023). Of these, MaxEnt has become the most widely used method in ecology as it employs a machine learning approach based on the principle of maximum entropy, using straightforward and precise mathematical formulas to predict species distributions. Additionally, it is valued for its ease of operation, rapid computation, and high accuracy and stability (Gao et al. 2025). The MaxEnt is particularly effective when working with incomplete, small‐sample, or discrete distribution data, enabling the reliable prediction of suitable habitats while maintaining strong interpretability (Elith et al. 2011). Indeed, the method has been widely utilized across plants, including species such as *Xerophyta* spp. (Wanga et al. 2024) and *Hibiscus mutabilis* (Zhang, Jiang, et al. 2024).

*Meconopsis* spp., commonly known as ‘Himalayan blue poppy’, are annual or perennial herbaceous plants in the Papaveraceae family. According to a 2014 report, 79 species have been reported worldwide, with 58 of these occurring in China, primarily across the Qinghai‐Tibet Plateau and its adjacent regions (Yu et al. 2020). However, this number may change and require validation as new taxa are discovered. The genus exemplifies co‐evolution between flora and environment, diverging through the distinctive geological and climatic processes of the Qinghai‐Tibet Plateau (Wang, Baskin, et al. 2018). For instance, cooling induced by its uplift promoted gradual adaptation to the cold, high‐altitude environments (Xie et al. 2014). However, increased global carbon emissions and the intensification of the greenhouse effect have recently posed challenges of deacclimation (Yu et al. 2020). In China, *Meconopsis* species are primarily distributed in high‐altitude regions of the southwest and west, serving as flagship plants in the Himalayan alpine zone. Although Previous modeling studies have primarily focused on predicting habitat suitability of *Meconopsis integrifolia* and *M. punicea* , data from the Global Biodiversity Information Facility (GBIF) show broad global occurrence of these species, limiting their representativeness for the genus under changing climates (Xiao 2013; Shi et al. 2022; Guo and Wang 2023). To address this research gap, we examined Seven additional species, which includ *Meconopsis betonicifolia* Franch, *Meconopsis delavayi* (Franch.) Franch. ex Prain, *Meconopsis henrici* Bureau & Franch, *Meconopsis lyrata* (H. A. Cummins & Prain) Fedde, *Meconopsis paniculata* (D. Don) Prain, *Meconopsis simplicifolia* (D. Don) Walp and *Meconopsis wilsonii* Gray‐Wilson (Raven and Wu 1994) from China (with ≥ 10 occurrence records) not previously documented in the literature. Here, using the MaxEnt approach, we aim to: (a) identify key environment variables influencing spatial patterns of these Seven *Meconopsis* spp.; (b) map current potential distribution across China; and (c) predict centroid shifts and habitat suitability under three future climate scenarios (50s and 70s). Findings from this study will provide critical data to support future seed conservation and reintroduction efforts for *Meconopsis* spp.

## 2 Methods

### 2.1 Species Occurrence Data

**FIGURE 1:**
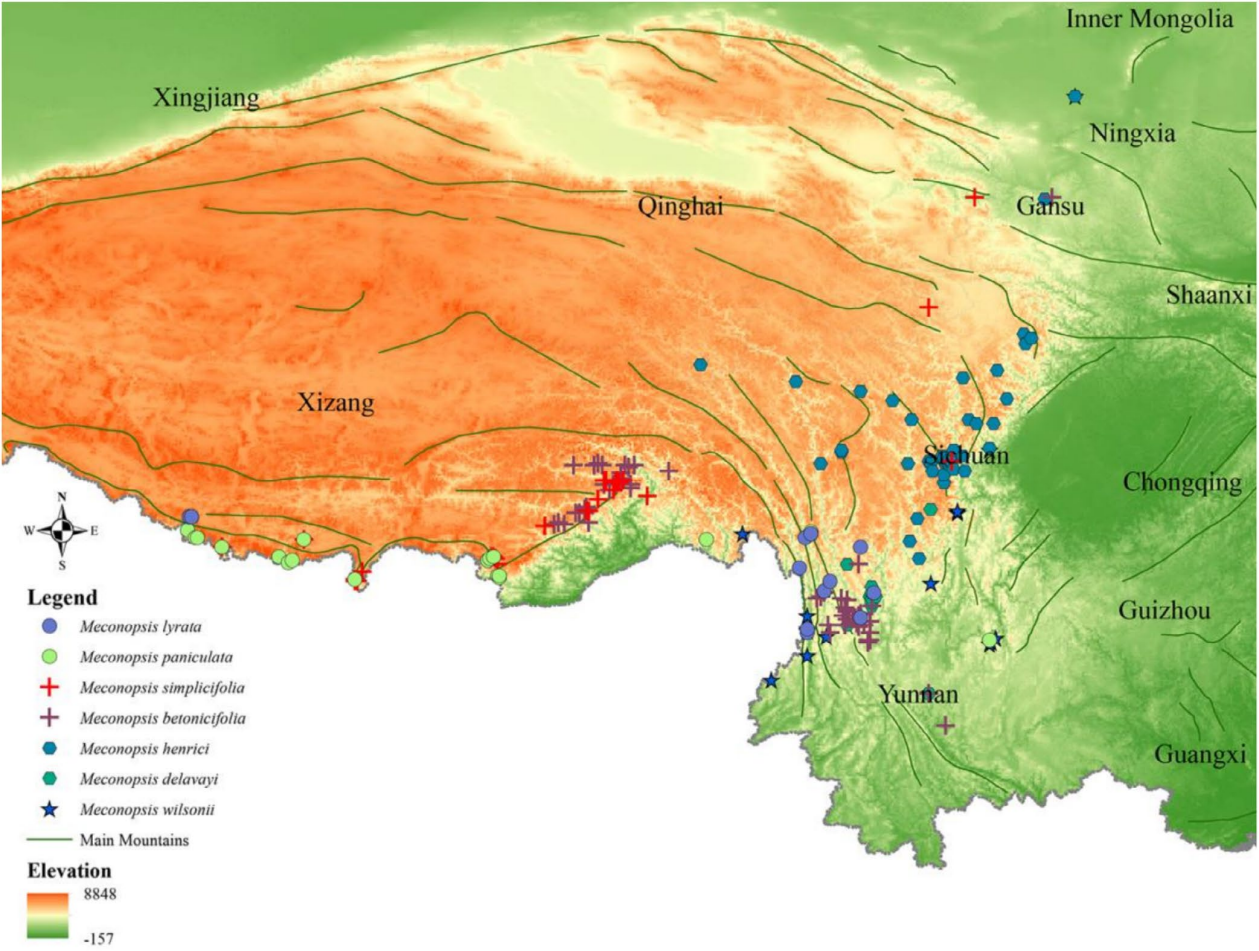
Locality and occurrence records of *Meconopsis* spp. employed in the modeling.

Occurrence records for seven *Meconopsis* species were obtained from our field investigations, as well as the Global Biodiversity Information Facility (GBIF, http://www.gbif.org/), the Chinese Virtual Herbarium (CVH, https://www.cvh.ac.cn/), and the National Specimen Information Infrastructure (NSII, https://www.nsii.org.cn/2017/), all accessed in March 2026. Duplicate, incomplete, and erroneous records were removed. We employed ENMTools 1.3 (http://purl.oclc.org/enmtools) with a 2.5‐min raster resolution to eliminate spatially redundant points (Warren et al. 2010). The cleaned datasets (Table S1, Table 1) were used for subsequent analyses. Species distribution maps were generated in ArcGIS 10.8 (Esri, Redlands, CA, USA; http://www.esri.com) (Figure 1).

**TABLE 1:** Valid occurrences of seven species.

Species	Occurrences
*Meconopsis betonicifolia*	64
*Meconopsis delavayi*	11
*Meconopsis henrici*	41
*Meconopsis lyrata*	12
*Meconopsis paniculata*	21
*Meconopsis simplicifolia*	27
*Meconopsis wilsonii*	15

### 2.2 Environmental Variables

Nineteen bioclimatic variables (Bio1‐Bio19) data were obtained from the WorldClim database (Version 2.1; https://www.worldclim.org/) (Fick and Hijmans 2017) for the baseline period (1970–2000) and future periods (2050s and 2070s) under the BCC‐CSM2‐MR, that is a climate model suited for China that was developed by the China Meteorological Administration (Gao et al. 2025), model with three SSP, which represents ‘Shared Socioeconomic Pathways’ scenarios (SSP126, SSP245, SSP585). SSP1‐2.6 is a sustainable development pathway with low carbon emissions, SSP2‐4.5 represent a middle‐of‐the‐road development pathway with moderate carbon emissions, and SSP5‐8.5 is a rapid development pathway with high carbon emissions. In addition, the mid‐century (2050s) and late‐century (2070s) were selected as two key future temporal nodes to obtain research data with greater timeliness and early warning value. Therefore, we chose these specific scenarios and two time points to simulate the suitable habitat distribution of *Meconopsis* spp. for the future time frame. All data were at 2.5 arc‐min spatial resolution. Raster layers were clipped to China's geographical boundaries in ArcGIS 10.8 and converted to ASCII format files, which only include letters, numbers and common symbols, for subsequent analysis and modeling. Furthermore, aspect, slope, and elevation data obtained from Geospatial Data Cloud (https://www.gscloud.cn/).

To minimize multicollinearity, we assessed correlations among the 22 bioclimatic variables in ENMTools v1.3 under current climate conditions, followed by a Maxent pre‐analysis. After, based on the pre‐analysis outputs, we ranked the variables by their percent contribution in descending order. We then selected the five environmental factors that exhibited the highest contributions while with pairwise correlation coefficients < 0.75. For this candidate set, we further calculated the variance inflation factor (VIF) and verified that all VIF values were < 10 (Table S2) so as to sufficiently reduce multicollinearity. If any retained variable still showed a high VIF, it was subsequently removed and replaced by the next most contributive variable that satisfied the correlation criterion. Ultimately, five reliable environmental predictors were identified for final model construction.

### 2.3 Model Optimization

We optimized species distribution and environmental data using the R package ENMeval (https://cran.r‐project.org/package = ENMeval). Regularization Multiplier (RM) was tested with a range of 0.5–4.0 in 0.5 increments, combined with six Feature Combination (FC) classes: L, LQ, H, LQH, LQHP, and LQHPT. Optimal parameter sets were selected based on ΔAICc values (i.e., ΔAICc = 0) (Burnham and Anderson 2004; Warren and Seifert 2011).

### 2.4 Species Distribution Modeling

Filtered environmental variables (ASCII format) were imported into MaxEnt version 3.4.1 (Phillips et al. 2026). For each species, the model was run 10 times with parameters defined by ENMeval optimization. Occurrence data were split into 75% training and 25% testing subsets (Li et al. 2020; Wanga et al. 2024). We used 10,000 random background points, with a maximum of 5000 iterations, logistic output, files saved as ASCII format, and the Jackknife method to assess variable contributions; all other settings remained default (Nzei et al. 2021). In our research, model performance was evaluated using the area under the ROC (receiver operating characteristic) curve (AUC) classified as: 0.5–0.6, failing; 0.6–0.7, poor; 0.7–0.8, general; 0.8–0.9, good; 0.9–1.0, very good (Swets 1988; Phillips et al. 2006). The AUC value ranges between 0 and 1, with values closer to 1 indicating superior model performance. Furthermore, True Skill Statistics (TSS) also were used to assess the accuracy of the MaxEnt models (Allouche et al. 2006; Warren and Seifert 2011). Final Seven *Meconopsis* spp. distribution predictions were based on the average across 10 replicates for three different climate change scenarios (SSP126, SSP245 and SSP585) in the 2050s or 2070s.

Predicted suitability maps were imported into ArcGIS 10.8 and classified into four categories: unsuitable (*p* < 0.24), low suitable (0.24 ≤ *p* < 0.33), moderately suitable (0.33 ≤ *p* < 0.48), and highly suitable areas (*p* ≥ 0.48) (Zhang et al. 2023). Area statistics were extracted using ArcGIS 10.8 for each suitability class.

### 2.5 Shifts in the Distribution Center of Suitable Habitats for *Meconopsis* spp.

Using current distribution as a baseline, we applied ArcGIS 10.8 spatial statistics to calculate changes in suitable habitat and track centroid shifts of the Seven *Meconopsis* spp. under future climate scenarios, thereby evaluating the potential migration route (Zhang, Jiang, et al. 2024).

## 3 Results

### 3.1 Variable Selection and Model Optimization

The top five contributing variables for each species, along with screened distribution data of Seven *Meconopsis* spp., were imported into the MaxEnt model. Delta AICc values for different parameter combinations were obtained using ENMeval (Figure 3). For all Seven species, delta AICc values under default parameters were either > 2 or null, indicating that default MaxEnt settings were unreliable. To improve model performance, two key parameters, regularization multiplier (RM) and feature combination (FC), were optimized based on ENMeval (Table 2). Model accuracy was assessed with ROC curve analysis. After 10 simulations, the AUC values for both training and test datasets exceeded 0.9 (Table 3) under current and future climate conditions, confirming that the optimized MaxEnt models produced reliable predictions and closely matched observed species distributions. The True Skill Statistic (TSS) also used to evaluate predictive performance of the MaxEnt models for the seven *Meconopsis* species. Overall, the models exhibited high accuracy and generally good stability, which TSS values ranged from 0.660 to 0.908 (Table 4), with *M. betonicifolia* achieving the highest performance (TSS = 0.908), followed by *M. henrici* (TSS = 0.848) and *M. paniculata* (TSS = 0.830).

**TABLE 2:** Optimal regularization multiplier and feature combination for maxent.

Species	RM	FC
*Meconopsis betonicifolia*	1.5	LQHPT
*Meconopsis delavayi*	1.5	LQ
*Meconopsis henrici*	0.5	LQ
*Meconopsis lyrata*	1.0	LQ
*Meconopsis paniculata*	1.5	LQH
*Meconopsis simplicifolia*	2.0	LQH
*Meconopsis wilsonii*	0.5	LQ

**TABLE 3:** Model performance evaluation for Seven Meconopsis species using Area Under the Curve (AUC) in Maxent.

Species	AUC	Standard deviation
*Meconopsis betonicifolia*	0.989	0.002
*Meconopsis delavayi*	0.991	0.005
*Meconopsis henrici*	0.976	0.009
*Meconopsis lyrata*	0.992	0.004
*Meconopsis paniculata*	0.996	0.001
*Meconopsis simplicifolia*	0.976	0.007
*Meconopsis wilsonii*	0.972	0.015

**TABLE 4:** Model performance evaluation for seven *Meconopsis* species using the True Skill Statistic (TSS).

Species	TSS
*Meconopsis betonicifolia*	0.908
*Meconopsis delavayi*	0.725
*Meconopsis henrici*	0.848
*Meconopsis lyrata*	0.660
*Meconopsis paniculata*	0.830
*Meconopsis simplicifolia*	0.795
*Meconopsis wilsonii*	0.711

### 3.2 Relationship Between Species Distribution and Environmental Variables

**FIGURE 2:**
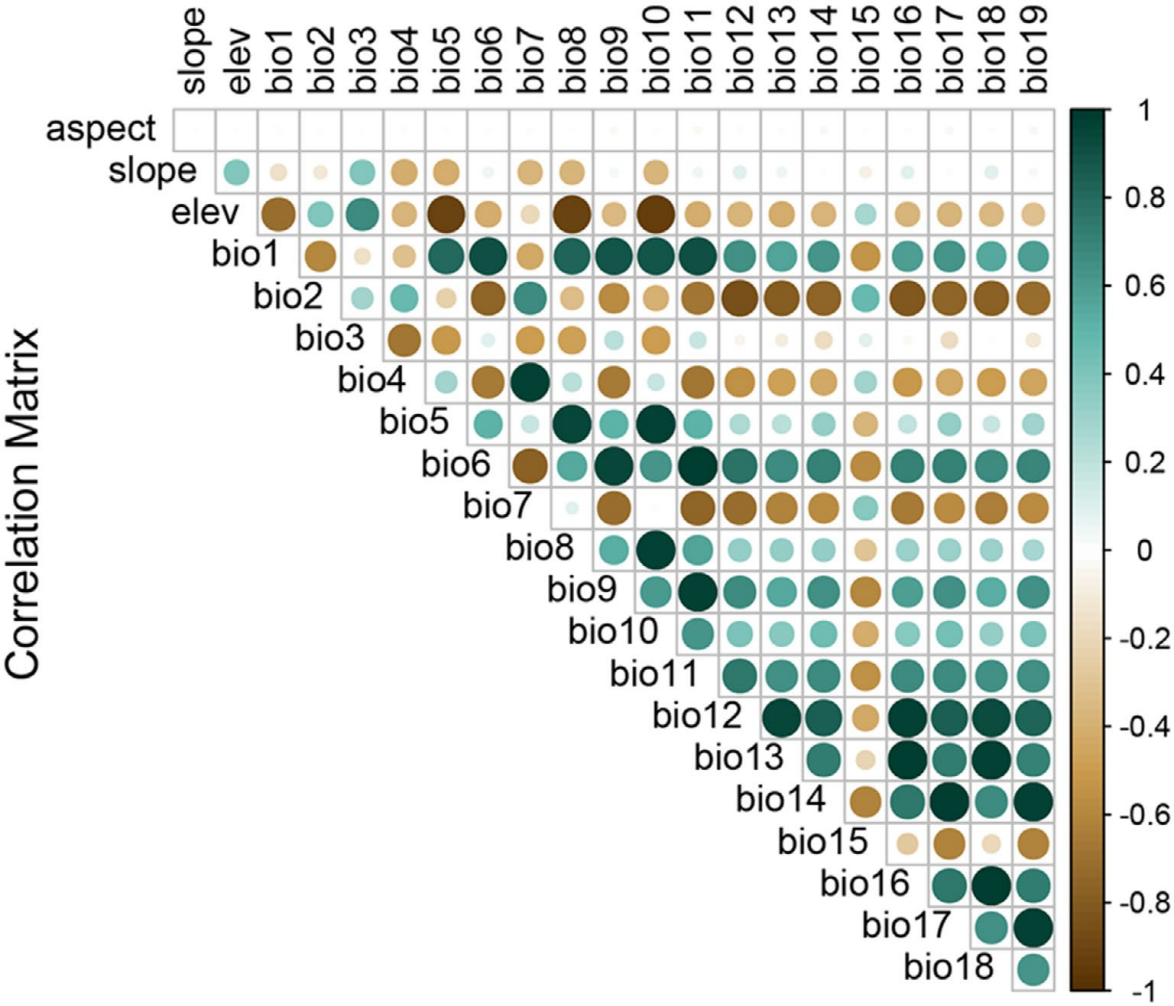
Correlation matrix of bio1‐19 and elevation.

From the correlation analysis of 19 climate variables and elevation (ENMTools; Figure 2) and MaxEnt pre‐analysis, five key variables were selected for simulation (Table 5). The dominant environmental drivers explaining species distribution varied among species. For example, Bio 4 (temperature seasonality (standard deviation × 100)) was the most important factor explaining the distribution of *M. betonicifolia* (49.6%). The distribution of *M. delavayi* was most explained by Bio 3 (Isothermality (Bio 2/Bio 7) (× 100)) (44.3%). *M. henrici* distribution was explained most by Bio11 (40.7%), whereas *M. lyrata* distribution was explained by Bio 4 (50.2%). Bio 3 explained the distribution of *M. paniculata* the most (32.3%), whereas Bio4 (31.0%) explained the distribution of *M. simplicifolia* the most. Finally, the distribution of *M. wilsonii* was explained by Slope (31.1%). Our Jackknife tests (Figure 4) demonstrated that three environmental variables (Bio 3, Bio 4, and elevation) were consistently the most influential predictors. Collectively, these three variables determined the potential distribution patterns of *Meconopsis* spp.

**TABLE 5:** Variable contribution to the final MaxEnt models of *Meconopsis* species.

Species	Variance				
*Meconopsis betonicifolia*	bio4 (49.6%)	elev (20.3%)	bio3	bio12	bio15
*Meconopsis delavayi*	bio3 (44.3%)	bio4 (32.0%)	bio16	bio15	elev
*Meconopsis henrici*	bio11 (40.7%)	bio3 (28.0%)	elev	bio12	aspect
*Meconopsis lyrata*	bio4 (50.2%)	bio15 (25.9%)	slope	elev	bio14
*Meconopsis paniculata*	bio3 (32.3%)	slope (25.9%)	bio11	bio19	bio10
*Meconopsis simplicifolia*	bio4 (31.0%)	elev (30.7%)	bio3	bio13	bio14
*Meconopsis wilsonii*	slope (31.1%)	bio3 (23.9%)	bio11	bio4	bio15

**FIGURE 3:**
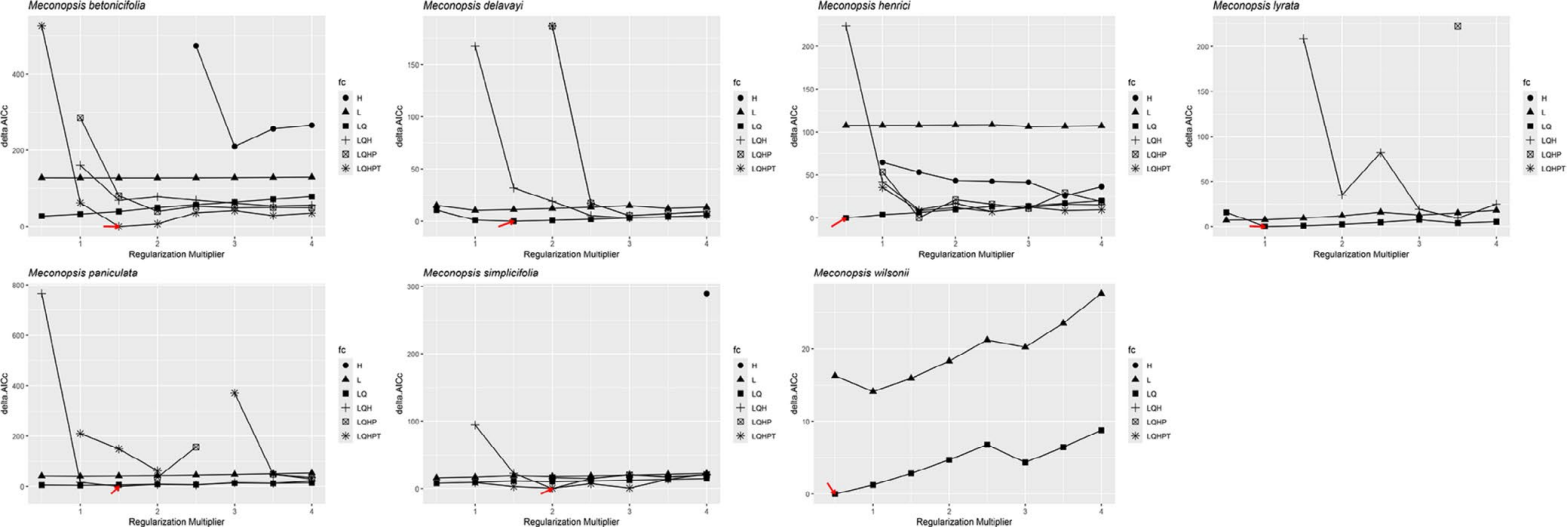
Delta AICc of MaxEnt models under different regularization multiplier and feature combination.

**FIGURE 4:**
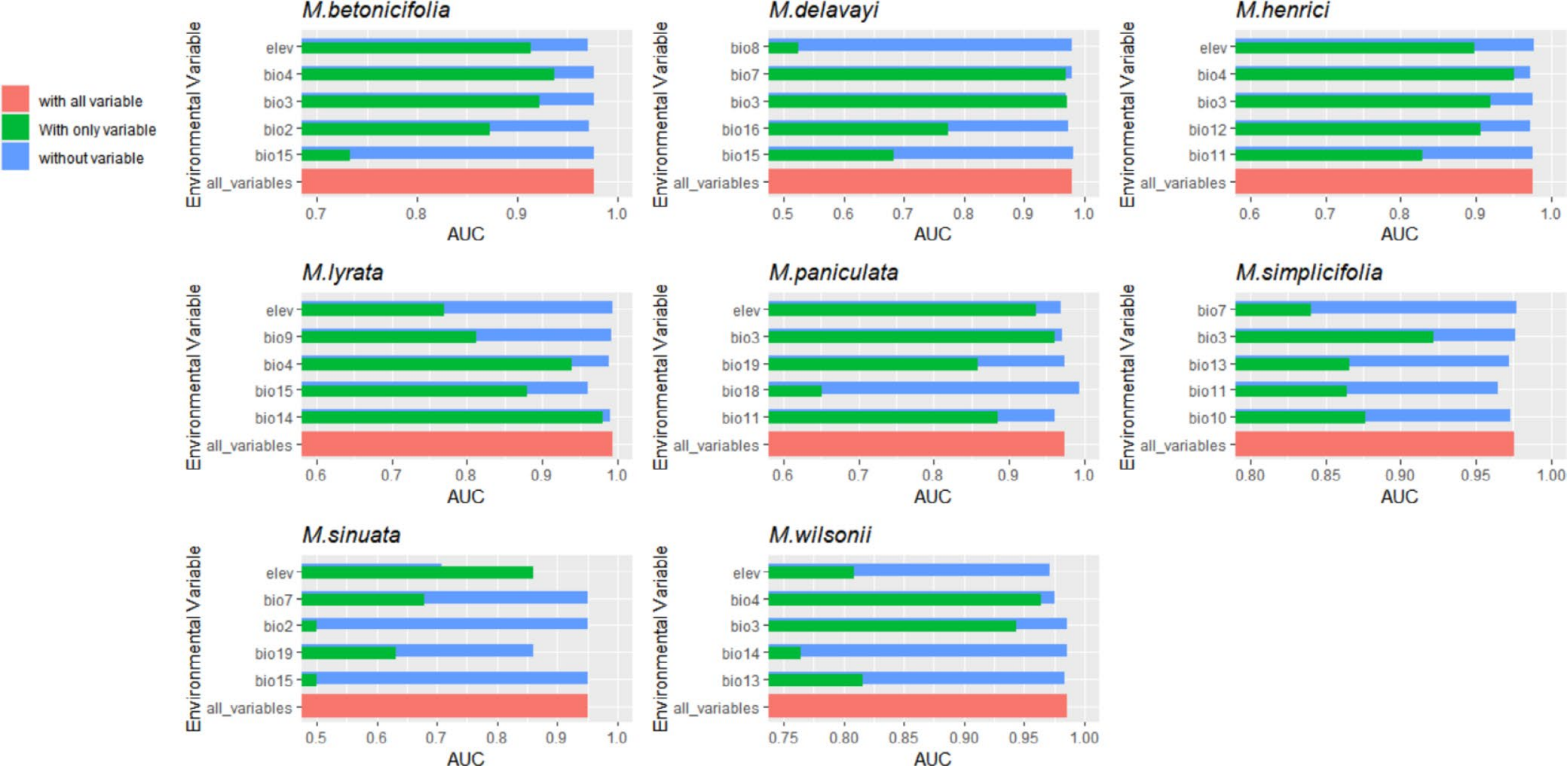
Jackknife results for *Meconopsis* spp. distribution using MaxEnt models.

**FIGURE 5:**
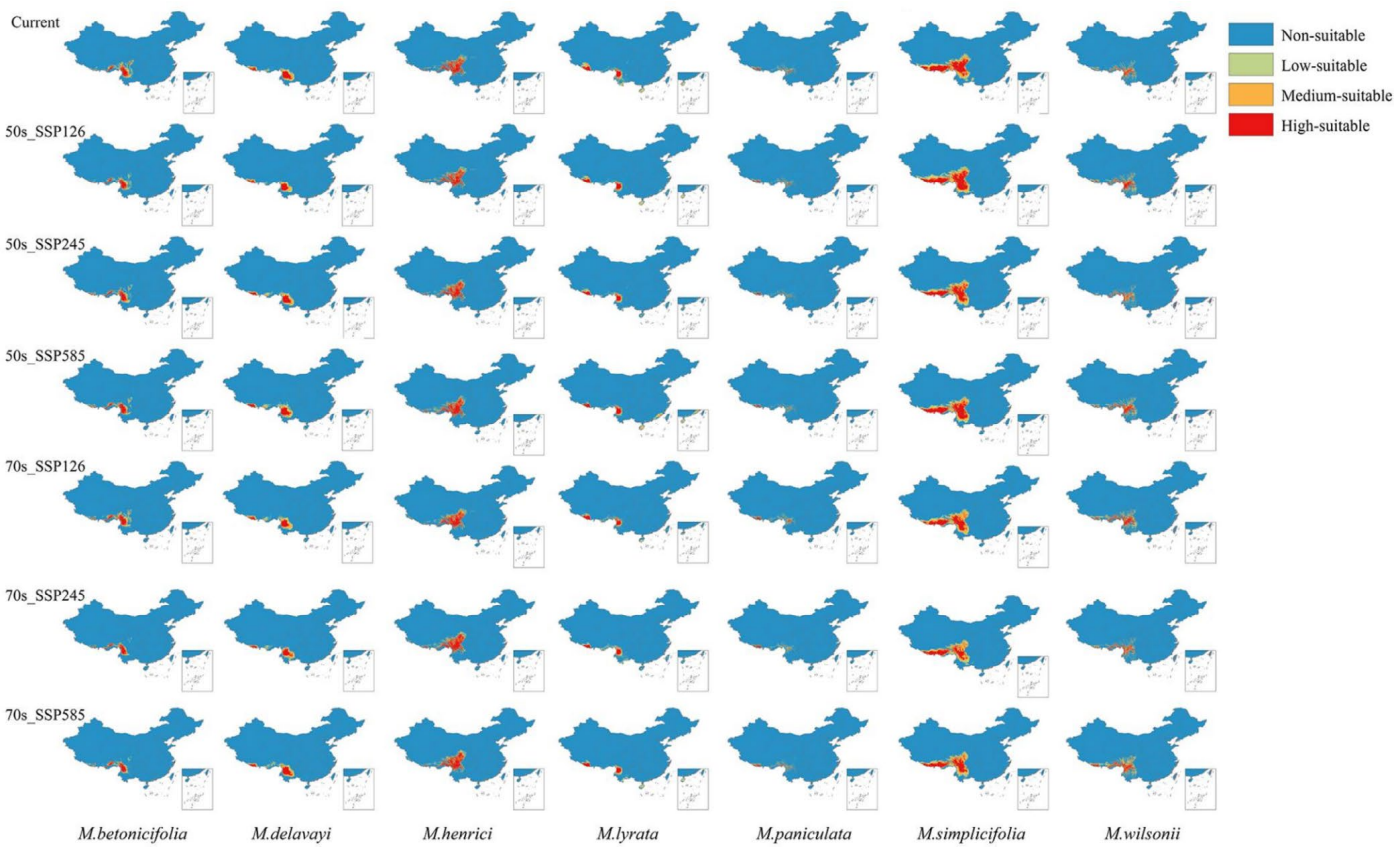
*Meconopsis* spp. prediction maps for the current, future climate, 50s‐SSP126, 50s‐SSP245, 50s‐SSP 585, 70s‐SSP126, 70s‐SSP245, and 70s‐SSP585.

**FIGURE 6:**
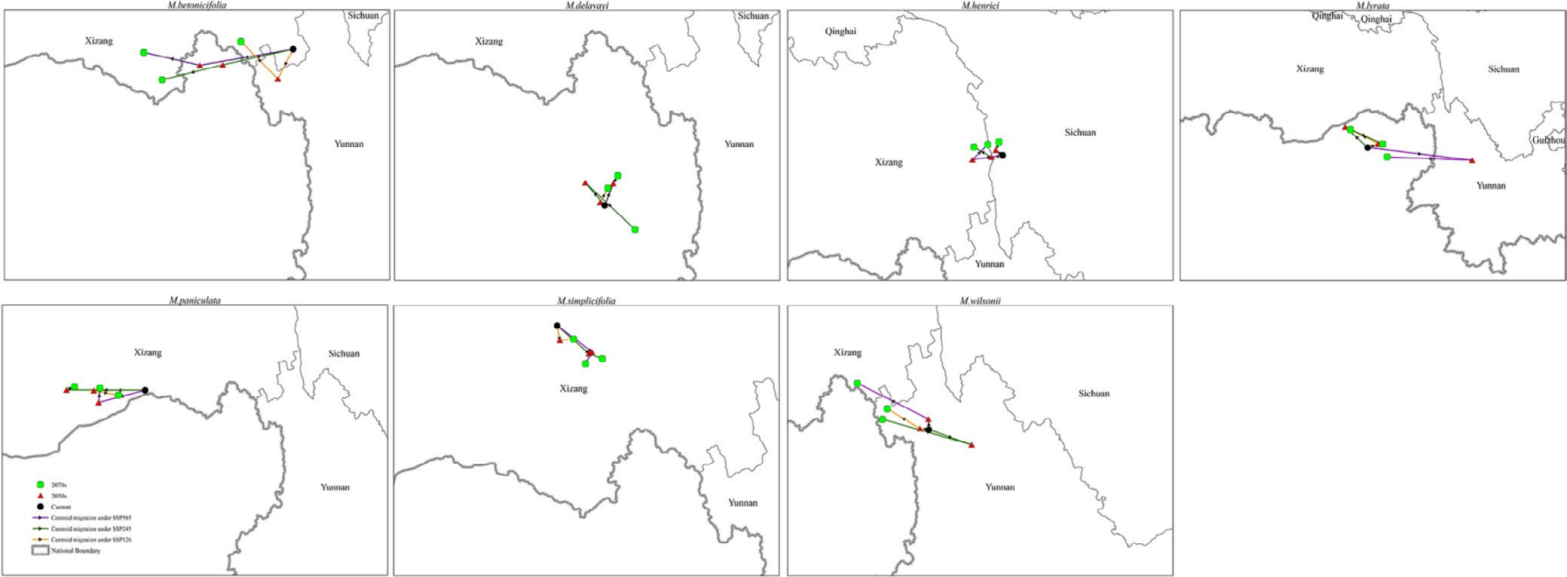
Centroid migration of *Meconopsis* spp. under future climate scenarios.

**FIGURE 7:**
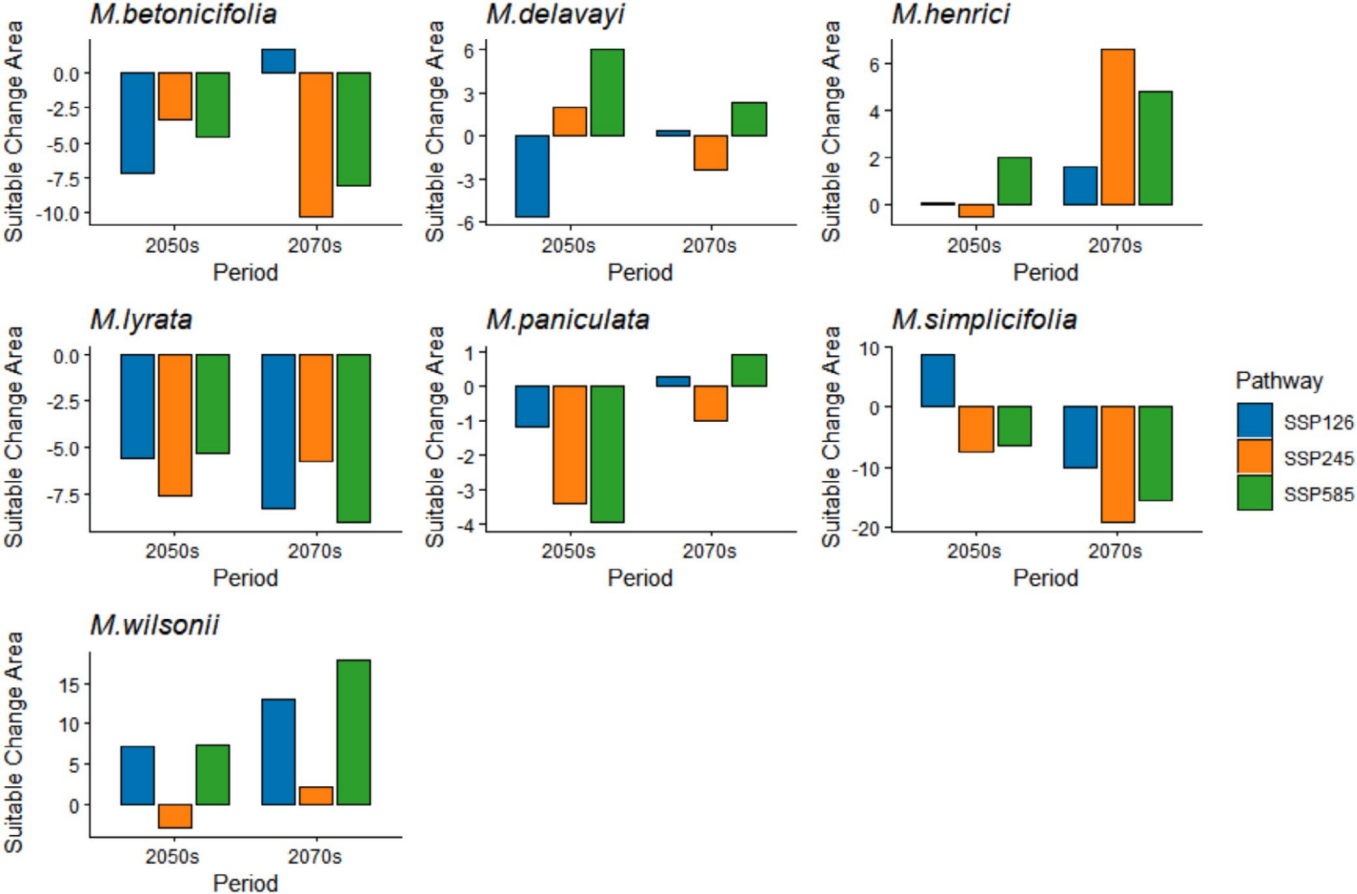
Suitable change area (×10^4^ km^2^) of *Meconopsis* spp. under the different pathway.

### 3.3 Distribution of Potential Suitable Habitats

MaxEnt models (Figure 5; Table 6) under the current climate conditions showed that high‐, medium‐, and low‐suitability areas across the Seven species ranged from 2.96–46.44 (×10^4^ km^2^), 4.60–29.52 (×10^4^ km^2^) and 4.53–20.80 (×10^4^ km^2^), respectively. Most species had suitable habitats concentrated in southwestern China, including Xizang, Sichuan, Yunnan, Guizhou, and Qinghai. For these species, total suitable habitat accounted for 1.3%–10.1% of land area in China, with high‐suitability zones making up 24.4%–48.0% of the total suitable area.

**TABLE 6:** Current suitable area (×10^4^ km^2^).

Species	Non‐suitable	Low‐suitable	Medium‐suitable	High‐suitable
*Meconopsis betonicifolia*	930.12	10.22	10.07	10.94
*Meconopsis delavayi*	931.88	7.87	9.93	11.66
*Meconopsis henrici*	911.41	10.38	13.73	23.09
*Meconopsis lyrata*	929.52	11.41	7.93	9.75
*Meconopsis paniculata*	946.52	4.53	4.60	2.96
*Meconopsis simplicifolia*	864.58	20.80	29.52	46.44
*Meconopsis wilsonii*	930.06	10.29	10.22	8.05

Under future climate scenarios, distribution trends varied among species (Figure 7). *M. lyrata* consistently lost suitable habitat under all carbon emission scenarios. Meanwhile, *M. betonicifolia* and *M. simplicifolia* contracted under five carbon emission scenarios. Conversely, *M. henrici* expanded under all carbon emission scenarios, except 2050s‐SSP245. Other species showed that for example, *M. wilsonii* shrunk under three climate change scenarios in the SSP245, but expanded in the SSP126 and SSP585, *M. paniculata* contracted in the 2050s and under SSP585 in the 2070s but expanded in all other cases.

### 3.4 Shifts in Distribution Centroids Under Climate Change

Our centroid analysis results showed clear spatial shifts in potential habitats (Figure 6). For *M. betonicifolia*, the centroid remained in near the Xizang‐Yunnan‐Myanmar shifting 39.56–101.73 km in various directions across climate scenarios. The distribution centroid of the species first moved 39.56 km southeast from the existing point (94 34′20.2″E, 28 20′35.8″ N), and then moved 60.01 km northwest under the SSP126. Under the SSP245 scenario, the center of mass first moved southeast by 70.54 km, then turned northwest by 67.56 km, and subsequently shifted to the location (97 08′28.5″E, 28 00′33.2″). Under the SSP585 scenario, the center of mass first moved southwest by 101.73 km, then moved northwest by 61.84 km, and eventually moved to 96 56′32.8″E, 28 18′31.5″ N. For *M. delavayi*, the center of mass remained in Myanmar, shifting 6.10‐78.42 km depending on climate change scenarios, with small shifts trend (< 35 km) except under the SSP245 scenario during the 2050s‐2070s. *M. henrici* distribution centroid centered around Xizang Autonomous Region and Sichuan Province, China, the distribution centroid is consistently farther north in the 2070s than in the 2050s, across all three climate scenario. The distribution centroid of *M. lyrata* lies on the Yunnan‐Myanmar border, with a maximum centroid displacement of approximately 458 km. Under the SSP126 scenario, the distribution centroid shifted from 95 48′81″E, 26 38′64″N, first northeastward and then northwestward, ultimately moving to 95 03′06″E, 27 25′43″N. Under the SSP245 scenario, the centroid will shift northwest first, then southeast, and finally to 96 28′47″E, 26 47′80″N in the 70s. Under the SSP585 scenario, its center of mass shifted east first, then west, and finally to 96 39′66″E, 26 14′24″N. The distribution centroid of *M. paniculata* was centered around the Xizang Autonomous Region, and across different SSP scenarios, it generally exhibited a westward shifting trend. The distribution center for *M. simplicifolia* was always located in Xizang Autonomous Region, China, with the movement of ~12.1–40.4 km, and the centroid of *M. simplicifolia* towards the southwest direction generally. Irrespective of scenario and era, the distribution center of *M. wilsonii* was almost always concentrated in northwestern Yunnan, except that under the SSP585 scenario in the 2070s, the distribution centroid was located in southeastern Xizang; across climate change scenarios, its centroid shifted by approximately 13–134 km. Overall, centroid shifts suggest strong regional variation in species responses, with expansions and contractions influenced primarily by temperature seasonality, isothermality, and elevation.

## 4 Discussion

### 4.1 MaxEnt Model Optimization

The MaxEnt model employs a complex machine learning algorithm that is sensitive to sampling bias and prone to overfitting. Predictions based on default parameters often yield low accuracy. Therefore, it is necessary to use the ENMeval package to adjust the RM and FC parameters, identify the optimal settings, and select models with lower complexity (Zhu and Qiao 2016). ENMeval has been widely used in species distribution studies, such as those on *Melliodendron xylocarpum* (Wang, Xu, and Li 2018), *Solanum rostratum* (Zhang, Sun, et al. 2024), and *Prunus mira* (Zhang, Bai, et al. 2024), where optimized models achieved higher predictive accuracy. Results from ENMeval evaluations showed that the model built with default parameters, FC (L + Q + P + H) and RM (1.0), was overly complex and poorly predictive. To address this, we evaluated and optimized the MaxEnt models for Seven *Meconopsis* spp., selecting low‐complexity models suitable for each species. In this study, the MaxEnt models we applied exhibited high AUC values and favorable TSS values, which is comparable to various studies done in the region using MaxEnt modeling approaches (Yang et al. 2020). However, limited by the number of occurrence records, the TSS values of *M. delavayi* (TSS = 0.725), *M. wilsonii* (TSS = 0.711), and *M. lyrata* (TSS = 0.660) still have room for improvement. In future research, we will further strengthen the collection of distribution data for these two species and improve the reliability of model predictions.

### 4.2 The Key Environment Variables Influencing Distribution of *Meconopsis* spp.

Species distribution is an important spatial feature, closely related to extinction risk, ecological invasions, and niche breadth. Under climate change, predicting potential habitats and identifying limiting factors provides a scientific basis for effective protection and sustainable use of plant resources (Zheng et al. 2023). Our study showed that while the dominant limiting factors differ among Seven *Meconopsis* spp., three variables, Bio3, Bio4, and elevation are the most influential. These variables appeared six, five, and five times, respectively, among the dominant factors, and Bio3, Bio4 as the most important limiting variables appeared twice, three times, separately. Previous studies confirm the importance of these factors, explaining the distribution of alpine plants. For example, Bio3 is one of the five important variables for the distribution of *M. integrifolia* (Guo and Wang 2023), while Bio4 contributed to the distribution of *M. punicea* on the Qinghai‐Tibet Plateau (Shi et al. 2022). Bio3 also contributed 44.5% to the distribution of *Castanopsis delavayi* (Zhang et al. 2021). Similarly, Li (2023) reported that Bio4 strongly influences the distribution of five *Sabina* species, including *S. vulgaris* , *S. pendula* , and *S. macrocarpa* in Xizang, with cumulative contributions above 70%. For the species *Quercus semecarpifolia*, Bio3, Bio4, and elevation jointly determine its distribution (Wang et al. 2023).

Although Bio3, Bio4, and elevation dominate the distribution of many alpine plants, each *Meconopsis* species also responds to unique environmental factors. Noticeably, precipitation related variables, including Slope, Bio11 (Mean Temperature of Coldest Quarter), Bio12 (annual precipitation), Bio13 (wettest monthly precipitation), Bio14 (driest month), Bio15 (coefficient of variation of precipitation), Bio16 (wettest quarter), and Bio19 (coldest quarter), also affect the distribution of *Meconopsis* spp., though less strongly. Similar findings have been reported for *Rhododendron* spp. (Li et al. 2023).

### 4.3 Future Changes in Suitable Habitats for *Meconopsis* spp.

Under the future climate scenarios, projected habitat shifts vary widely among species. The potential suitable areas for *M. lyrata* show declining trends, indicating possible population contractions in the future. Other species displayed inconsistent patterns across timeframes and emission scenarios. These differences reflect species‐specific adaptive capacities. For example, *Viburnum keteleeri* and *V. opulus* show strong phenological plasticity, while *V. carlesii* exhibits little plasticity (Xu 2017). Consistently, in this study, the diverse genus *Meconopsis* demonstrates varying responses to environmental change, underscoring its ecological diversity.

Centroids' distribution of all Seven *Meconopsis* species revealed no large‐scale spatial shifts in species distributions, suggesting overall stability, though localized changes were random and lacked clear trends. While global warming is expected to increase both temperature and precipitation (Jiang et al. 2008), the interactions among 19 climate variables are highly complex (Guo et al. 2022; Black 2024; Lu et al. 2024), making it difficult to predict uniform habitat responses across species.

### 4.4 Strategies for *Meconopsis* Conservation

For larger‐biomass plants, rainfall is often the dominant factor, whereas smaller biomass species are more strongly driven by temperature (Yang et al. 2024). As small alpine herbs, *Meconopsis* species are primarily influenced by temperature‐related variables, especially isothermality and seasonality. With the warming climate, some species of *Meconopsis* may expand and could be incorporated into urban horticulture, while others are likely to contract. For declining species, ex‐situ conservation efforts, particularly through institutions like the National Botanical Garden, are critical. These efforts should prioritize collection, cultivation, and propagation using advanced facilities, ensuring the preservation of *Meconopsis* diversity. And, we considered that when conserving these alpine species in lower‐altitude regions, cryogenic facilities can be utilized, such as artificial climate chambers. Furthermore, we also suggest that the development of a high‐altitude genetic resource center should be provided for the conservation of *Meconopsis* spp.

### 4.5 The Potential Limitations and Uncertainties

Although we obtained species distribution data from the GBIF, the CVH, the NSII, as well as our own field surveys, these data are still subject to sampling biases, resulting in spatial and temporal unevenness. Therefore, in subsequent studies, we will further acquire more comprehensive species distribution data through field surveys and data retrieval. Moreover, ecological niche modeling is a useful tool predicting the potential impacts of climate change on both species distributions and their responses (Ngarega et al. 2022). Generally, multiple Global Climate Model (GCM) would typically provide more robust and defensible results, while one GCM for future climate projections may not fully capture the actual distributions of species. Although we prioritized the BCC‐CSM2‐MR model in this study, a climate model well suited for predicting species distributions in China that also provides relatively reliable projections (Gao et al. 2025; Zheng et al. 2023). BCC‐CSM2‐MR may not fully represent the ensemble spread of available climate models. Therefore, in future work, we would integrate multiple GCM to enhance the stability and reliability of the analysis. On the other hand, although Bio3, Bio4, and elevation were identified as the dominant driving factors, different species exhibited divergent responses including range expansion, stable distribution, and range contraction under future climate emission scenarios. Such differences are mainly attributed to the inherent climate adaptation strategies of each species. Species differ substantially in their niche breadth along these key environmental gradients, which consequently results in their distinct sensitivity and adaptability to environmental changes. The core focus of this study is placed on the contribution analysis of environmental variables, species response curves, and spatiotemporal dynamics of suitable habitats. We considered that in‐depth ecological interpretation of how changes in key environmental factors specifically threaten species is future research. And we will further explore this issue quantitatively by calculating niche breadth and niche overlap to elaborate how the variation of critical environmental factors affects threatened species.

## 5 Conclusions

In this study, using the MaxEnt model, we modeled the current and future distribution of seven *Meconopsis* species under various future climate change scenarios. Our findings highlight Bio3, Bio4, and elevation as the key determinants of *Meconopsis* distribution. Climate change is projected to cause disproportionate effects on *Meconopsis* distribution, with some species experiencing range contractions, while others may expand across China. These results emphasize the need for species‐specific conservation strategies. Future research integrating ecology, genomics, and phylogeography will be essential to deepen our understanding of the evolution and ecological adaptation of *Meconopsis* in China and across Asia. In addition, we should adapt different strategies for *Meconopsis* conservation, according to climate changes in the future.

## Author Contributions

**Yuanzhao Yang:** conceptualization (lead), data curation (lead), software (lead), writing – original draft (lead), writing – review and editing (supporting). **Jiao Ma:** investigation (lead). **Xinyu Chen:** data curation (supporting), investigation (supporting). **Lu Zhang:** data curation (supporting), software (supporting). **Pei Tu:** formal analysis (lead). **Yuanzhi Pan:** writing – review and editing (lead).

## Funding

This work is supported by the Funding for the Chengdu Municipal Park City Construction and Management Bureau (202511KY0008).

## Conflicts of Interest

The authors declare no conflicts of interest.

## Supporting information


**Table S1:** Occurrence of all species.

**Table S2:** The VIF of seven *Meconopsis* species.

## Data Availability

Species occurrence data are available from Global Biodiversity Information Facility (GBIF, http://www.gbif.org/), the Chinese Virtual Herbarium (CVH, https://www.cvh.ac.cn/), and the National Specimen Information Infrastructure (NSII, https://www.nsii.org.cn/2017/). Climate dataare available from WorldClim (https://www.worldclim.org/). Aspect, slope, and elevation data obtained from Geospatial Data Cloud (https://www.gscloud.cn/).
