# Supplementary material for: The Impact of Climate Change on the Spatial Distribution of Seven Meconopsis Species in China: A MaxEnt Model‐Based Predictive Analysis
Source: Ecol Evol. 2026 Jun 17;16(6):e73824. doi: 10.1002/ece3.73824 (PMC13275543; doi:10.1002/ece3.73824)
Supplement: Supplementary file 1 — Table S1: Occurrence of all species. [file ECE3-16-e73824-s001.docx]

Table S1 Occurrence of all species

| Species | decimalLongitude | decimalLatitude |
| --- | --- | --- |
| *Meconopsis betonicifolia* | 100.0625 | 26.47916667 |
| *Meconopsis betonicifolia* | 100.1458333 | 26.10416667 |
| *Meconopsis betonicifolia* | 100.1458333 | 26.14583333 |
| *Meconopsis betonicifolia* | 100.1875 | 26.14583333 |
| *Meconopsis betonicifolia* | 100.1875 | 26.3125 |
| *Meconopsis betonicifolia* | 100.1875 | 26.5625 |
| *Meconopsis betonicifolia* | 100.2291667 | 26.89583333 |
| *Meconopsis betonicifolia* | 101.4791667 | 24.97916667 |
| *Meconopsis betonicifolia* | 101.8541667 | 24.27083333 |
| *Meconopsis betonicifolia* | 104.1875 | 35.85416667 |
| *Meconopsis betonicifolia* | 91.85416667 | 27.9375 |
| *Meconopsis betonicifolia* | 91.9375 | 27.97916667 |
| *Meconopsis betonicifolia* | 93.27083333 | 28.6875 |
| *Meconopsis betonicifolia* | 93.35416667 | 28.72916667 |
| *Meconopsis betonicifolia* | 93.47916667 | 28.6875 |
| *Meconopsis betonicifolia* | 93.6875 | 29.97916667 |
| *Meconopsis betonicifolia* | 93.72916667 | 28.9375 |
| *Meconopsis betonicifolia* | 93.8125 | 28.9375 |
| *Meconopsis betonicifolia* | 93.9375 | 28.9375 |
| *Meconopsis betonicifolia* | 93.9375 | 29.02083333 |
| *Meconopsis betonicifolia* | 93.97916667 | 29.0625 |
| *Meconopsis betonicifolia* | 94.02083333 | 28.72916667 |
| *Meconopsis betonicifolia* | 94.02083333 | 28.97916667 |
| *Meconopsis betonicifolia* | 94.14583333 | 29.97916667 |
| *Meconopsis betonicifolia* | 94.22916667 | 29.22916667 |
| *Meconopsis betonicifolia* | 94.22916667 | 30.02083333 |
| *Meconopsis betonicifolia* | 94.3125 | 29.97916667 |
| *Meconopsis betonicifolia* | 94.35416667 | 29.5625 |
| *Meconopsis betonicifolia* | 94.35416667 | 29.64583333 |
| *Meconopsis betonicifolia* | 94.39583333 | 29.64583333 |
| *Meconopsis betonicifolia* | 94.47916667 | 29.4375 |
| *Meconopsis betonicifolia* | 94.5625 | 29.4375 |
| *Meconopsis betonicifolia* | 94.5625 | 29.5625 |
| *Meconopsis betonicifolia* | 94.60416667 | 29.5625 |
| *Meconopsis betonicifolia* | 94.64583333 | 29.60416667 |
| *Meconopsis betonicifolia* | 94.72916667 | 29.60416667 |
| *Meconopsis betonicifolia* | 94.72916667 | 29.6875 |
| *Meconopsis betonicifolia* | 94.72916667 | 29.8125 |
| *Meconopsis betonicifolia* | 94.8125 | 29.97916667 |
| *Meconopsis betonicifolia* | 94.89583333 | 29.9375 |
| *Meconopsis betonicifolia* | 94.9375 | 29.47916667 |
| *Meconopsis betonicifolia* | 94.9375 | 29.5625 |
| *Meconopsis betonicifolia* | 95.02083333 | 29.97916667 |
| *Meconopsis betonicifolia* | 95.77083333 | 29.85416667 |
| *Meconopsis betonicifolia* | 99.02083333 | 27.0625 |
| *Meconopsis betonicifolia* | 99.10416667 | 27.10416667 |
| *Meconopsis betonicifolia* | 99.1875 | 27.1875 |
| *Meconopsis betonicifolia* | 99.27083333 | 26.47916667 |
| *Meconopsis betonicifolia* | 99.3125 | 26.3125 |
| *Meconopsis betonicifolia* | 99.5625 | 27.0625 |
| *Meconopsis betonicifolia* | 99.60416667 | 26.6875 |
| *Meconopsis betonicifolia* | 99.64583333 | 26.47916667 |
| *Meconopsis betonicifolia* | 99.64583333 | 26.85416667 |
| *Meconopsis betonicifolia* | 99.6875 | 26.60416667 |
| *Meconopsis betonicifolia* | 99.6875 | 26.77083333 |
| *Meconopsis betonicifolia* | 99.6875 | 26.8125 |
| *Meconopsis betonicifolia* | 99.6875 | 27.02083333 |
| *Meconopsis betonicifolia* | 99.72916667 | 26.6875 |
| *Meconopsis betonicifolia* | 99.8125 | 26.6875 |
| *Meconopsis betonicifolia* | 99.89583333 | 26.52083333 |
| *Meconopsis betonicifolia* | 99.9375 | 26.4375 |
| *Meconopsis betonicifolia* | 99.9375 | 26.6875 |
| *Meconopsis betonicifolia* | 99.9375 | 27.8125 |
| *Meconopsis betonicifolia* | 99.97916667 | 26.72916667 |
| *Meconopsis delavayi* | 100.1875 | 27.10416667 |
| *Meconopsis delavayi* | 100.2291667 | 26.89583333 |
| *Meconopsis delavayi* | 100.2291667 | 26.97916667 |
| *Meconopsis delavayi* | 100.2291667 | 27.0625 |
| *Meconopsis delavayi* | 100.2291667 | 27.1875 |
| *Meconopsis delavayi* | 100.2291667 | 27.3125 |
| *Meconopsis delavayi* | 100.3125 | 27.10416667 |
| *Meconopsis delavayi* | 101.4791667 | 24.97916667 |
| *Meconopsis delavayi* | 101.5208333 | 29.02083333 |
| *Meconopsis delavayi* | 99.6875 | 26.47916667 |
| *Meconopsis delavayi* | 99.6875 | 27.8125 |
| *Meconopsis henrici* | 100.6875 | 31.39583333 |
| *Meconopsis henrici* | 101.0208333 | 30.02083333 |
| *Meconopsis henrici* | 101.0625 | 28.3125 |
| *Meconopsis henrici* | 101.1041667 | 30.97916667 |
| *Meconopsis henrici* | 101.2291667 | 28.8125 |
| *Meconopsis henrici* | 101.2708333 | 27.9375 |
| *Meconopsis henrici* | 101.4791667 | 30.0625 |
| *Meconopsis henrici* | 101.5208333 | 30.02083333 |
| *Meconopsis henrici* | 101.5625 | 29.85416667 |
| *Meconopsis henrici* | 101.7708333 | 30.1875 |
| *Meconopsis henrici* | 101.8125 | 29.60416667 |
| *Meconopsis henrici* | 101.8125 | 29.77083333 |
| *Meconopsis henrici* | 101.8125 | 30.0625 |
| *Meconopsis henrici* | 101.8125 | 30.10416667 |
| *Meconopsis henrici* | 101.8125 | 30.14583333 |
| *Meconopsis henrici* | 101.9375 | 29.97916667 |
| *Meconopsis henrici* | 101.9375 | 30.0625 |
| *Meconopsis henrici* | 101.9375 | 30.10416667 |
| *Meconopsis henrici* | 101.9791667 | 30.0625 |
| *Meconopsis henrici* | 102.0208333 | 29.89583333 |
| *Meconopsis henrici* | 102.0208333 | 30.3125 |
| *Meconopsis henrici* | 102.2291667 | 30.1875 |
| *Meconopsis henrici* | 102.2291667 | 31.89583333 |
| *Meconopsis henrici* | 102.2708333 | 29.85416667 |
| *Meconopsis henrici* | 102.3541667 | 30.97916667 |
| *Meconopsis henrici* | 102.5208333 | 30.89583333 |
| *Meconopsis henrici* | 102.8125 | 30.35416667 |
| *Meconopsis henrici* | 102.8958333 | 30.89583333 |
| *Meconopsis henrici* | 102.9791667 | 32.0625 |
| *Meconopsis henrici* | 103.1875 | 31.4375 |
| *Meconopsis henrici* | 103.5625 | 32.85416667 |
| *Meconopsis henrici* | 103.6041667 | 32.64583333 |
| *Meconopsis henrici* | 103.7291667 | 32.77083333 |
| *Meconopsis henrici* | 104.0208333 | 35.8125 |
| *Meconopsis henrici* | 104.6875 | 38.0625 |
| *Meconopsis henrici* | 96.47916667 | 32.1875 |
| *Meconopsis henrici* | 98.5625 | 31.8125 |
| *Meconopsis henrici* | 99.10416667 | 30.02083333 |
| *Meconopsis henrici* | 99.5625 | 30.27083333 |
| *Meconopsis henrici* | 99.5625 | 30.3125 |
| *Meconopsis henrici* | 99.97916667 | 31.60416667 |
| *Meconopsis lyrata* | 100.2708333 | 27.1875 |
| *Meconopsis lyrata* | 85.27083333 | 28.85416667 |
| *Meconopsis lyrata* | 85.3125 | 28.85416667 |
| *Meconopsis lyrata* | 98.64583333 | 27.72916667 |
| *Meconopsis lyrata* | 98.77083333 | 28.39583333 |
| *Meconopsis lyrata* | 98.8125 | 26.3125 |
| *Meconopsis lyrata* | 98.8125 | 26.39583333 |
| *Meconopsis lyrata* | 98.89583333 | 28.47916667 |
| *Meconopsis lyrata* | 99.1875 | 27.22916667 |
| *Meconopsis lyrata* | 99.3125 | 27.4375 |
| *Meconopsis lyrata* | 99.97916667 | 26.64583333 |
| *Meconopsis lyrata* | 99.97916667 | 28.1875 |
| *Meconopsis paniculata* | 102.8125 | 26.14583333 |
| *Meconopsis paniculata* | 85.22916667 | 28.5625 |
| *Meconopsis paniculata* | 85.27083333 | 28.85416667 |
| *Meconopsis paniculata* | 85.3125 | 28.85416667 |
| *Meconopsis paniculata* | 85.39583333 | 28.39583333 |
| *Meconopsis paniculata* | 85.4375 | 28.39583333 |
| *Meconopsis paniculata* | 85.97916667 | 28.1875 |
| *Meconopsis paniculata* | 87.22916667 | 27.97916667 |
| *Meconopsis paniculata* | 87.4375 | 27.85416667 |
| *Meconopsis paniculata* | 87.52083333 | 27.89583333 |
| *Meconopsis paniculata* | 87.77083333 | 28.35416667 |
| *Meconopsis paniculata* | 88.89583333 | 27.47916667 |
| *Meconopsis paniculata* | 91.8125 | 27.89583333 |
| *Meconopsis paniculata* | 91.85416667 | 27.9375 |
| *Meconopsis paniculata* | 91.9375 | 27.97916667 |
| *Meconopsis paniculata* | 92.02083333 | 27.5625 |
| *Meconopsis paniculata* | 92.0625 | 27.52083333 |
| *Meconopsis paniculata* | 92.0625 | 27.5625 |
| *Meconopsis paniculata* | 96.60416667 | 28.35416667 |
| *Meconopsis paniculata* | 98.89583333 | 28.47916667 |
| *Meconopsis paniculata* | 98.89583333 | 28.52083333 |
| *Meconopsis simplicifolia* | 101.4791667 | 33.4375 |
| *Meconopsis simplicifolia* | 101.9791667 | 30.0625 |
| *Meconopsis simplicifolia* | 102.4791667 | 35.85416667 |
| *Meconopsis simplicifolia* | 85.97916667 | 28.1875 |
| *Meconopsis simplicifolia* | 85.97916667 | 28.1875 |
| *Meconopsis simplicifolia* | 87.77083333 | 28.35416667 |
| *Meconopsis simplicifolia* | 88.89583333 | 27.4375 |
| *Meconopsis simplicifolia* | 88.89583333 | 27.47916667 |
| *Meconopsis simplicifolia* | 88.9375 | 27.4375 |
| *Meconopsis simplicifolia* | 89.0625 | 27.64583333 |
| *Meconopsis simplicifolia* | 91.89583333 | 27.97916667 |
| *Meconopsis simplicifolia* | 91.9375 | 27.97916667 |
| *Meconopsis simplicifolia* | 91.97916667 | 27.9375 |
| *Meconopsis simplicifolia* | 92.02083333 | 27.8125 |
| *Meconopsis simplicifolia* | 93.0625 | 28.64583333 |
| *Meconopsis simplicifolia* | 94.02083333 | 28.97916667 |
| *Meconopsis simplicifolia* | 93.97916667 | 28.97916667 |
| *Meconopsis simplicifolia* | 94.22916667 | 29.22916667 |
| *Meconopsis simplicifolia* | 94.35416667 | 29.64583333 |
| *Meconopsis simplicifolia* | 94.39583333 | 29.64583333 |
| *Meconopsis simplicifolia* | 94.5625 | 29.4375 |
| *Meconopsis simplicifolia* | 94.60416667 | 29.60416667 |
| *Meconopsis simplicifolia* | 94.64583333 | 29.60416667 |
| *Meconopsis simplicifolia* | 94.64583333 | 29.64583333 |
| *Meconopsis simplicifolia* | 94.6875 | 29.60416667 |
| *Meconopsis simplicifolia* | 94.77083333 | 29.64583333 |
| *Meconopsis simplicifolia* | 95.3125 | 29.3125 |
| *Meconopsis wilsonii* | 101.5208333 | 27.39583333 |
| *Meconopsis wilsonii* | 102.1041667 | 28.9375 |
| *Meconopsis wilsonii* | 102.1041667 | 28.97916667 |
| *Meconopsis wilsonii* | 102.8125 | 26.0625 |
| *Meconopsis wilsonii* | 102.8125 | 30.3125 |
| *Meconopsis wilsonii* | 102.8541667 | 26.10416667 |
| *Meconopsis wilsonii* | 102.9375 | 26.1875 |
| *Meconopsis wilsonii* | 104.6875 | 38.0625 |
| *Meconopsis wilsonii* | 97.39583333 | 28.47916667 |
| *Meconopsis wilsonii* | 98.02083333 | 25.27083333 |
| *Meconopsis wilsonii* | 98.8125 | 25.8125 |
| *Meconopsis wilsonii* | 98.8125 | 26.35416667 |
| *Meconopsis wilsonii* | 98.8125 | 26.39583333 |
| *Meconopsis wilsonii* | 98.8125 | 26.6875 |
| *Meconopsis wilsonii* | 99.22916667 | 26.22916667 |
