# Supplementary material for: The Impact of Climate Change on the Spatial Distribution of Seven Meconopsis Species in China: A MaxEnt Model‐Based Predictive Analysis
Source: Ecol Evol. 2026 Jun 17;16(6):e73824. doi: 10.1002/ece3.73824 (PMC13275543; doi:10.1002/ece3.73824)
Supplement: Supplementary file 2 — Table S2: The VIF of seven Meconopsis species. [file ECE3-16-e73824-s002.docx]

Table S2 The VIF of seven *Meconopsis* species

| **Species** | **Variables** | **VIF** |
| --- | --- | --- |
| *Meconopsis betonicifolia* | bio12 | 4.27589 |
| *Meconopsis betonicifolia* | bio15 | 1.400299 |
| *Meconopsis betonicifolia* | bio3 | 3.696101 |
| *Meconopsis betonicifolia* | bio4 | 6.808133 |
| *Meconopsis betonicifolia* | elev | 3.037278 |
| *Meconopsis delavayi* | bio15 | 1.557035 |
| *Meconopsis delavayi* | bio16 | 3.456423 |
| *Meconopsis delavayi* | bio3 | 3.423763 |
| *Meconopsis delavayi* | bio4 | 6.031737 |
| *Meconopsis delavayi* | elev | 3.164013 |
| *Meconopsis henrici* | aspect | 1.007086 |
| *Meconopsis henrici* | bio11 | 3.967949 |
| *Meconopsis henrici* | bio12 | 2.522052 |
| *Meconopsis henrici* | bio3 | 3.581839 |
| *Meconopsis henrici* | elev | 4.092714 |
| *Meconopsis lyrata* | bio14 | 2.54565 |
| *Meconopsis lyrata* | bio15 | 1.61313 |
| *Meconopsis lyrata* | bio4 | 2.324145 |
| *Meconopsis lyrata* | elev | 2.118848 |
| *Meconopsis lyrata* | slpoe | 1.312307 |
| *Meconopsis paniculata* | bio10 | 5.679077 |
| *Meconopsis paniculata* | bio11 | 6.385254 |
| *Meconopsis paniculata* | bio19 | 2.349957 |
| *Meconopsis paniculata* | bio3 | 3.245452 |
| *Meconopsis paniculata* | slpoe | 1.339913 |
| *Meconopsis simplicifolia* | bio13 | 3.116749 |
| *Meconopsis simplicifolia* | bio14 | 3.05847 |
| *Meconopsis simplicifolia* | bio3 | 3.992798 |
| *Meconopsis simplicifolia* | bio4 | 5.537055 |
| *Meconopsis simplicifolia* | elev | 2.745742 |
| *Meconopsis wilsonii* | bio11 | 3.045052 |
| *Meconopsis wilsonii* | bio15 | 1.519957 |
| *Meconopsis wilsonii* | bio3 | 2.563161 |
| *Meconopsis wilsonii* | bio4 | 4.785657 |
| *Meconopsis wilsonii* | slpoe | 1.353119 |
